# Chronic pain in patients with gunshot wounds

**DOI:** 10.1186/s12871-023-02005-3

**Published:** 2023-02-07

**Authors:** Iurii Kuchyn, Vasyl’ Horoshko

**Affiliations:** National Medical University Named After O. O. Bogomolets, Kiev, Ukraine

**Keywords:** Chronic pain, Gunshot wounds, Pain, Pain management

## Abstract

**Background:**

In civilian life, from 11 to 40% of patients suffer from chronic pain after receiving injuries. There are almost no data on chronic pain in patients with gunshot wounds, isolated clinical cases have been published. The purpose of our study is to determine the factors that can potentially affect the results of treatment of such patients, namely the frequency of development of chronic pain, acute stress reactions, satisfaction with the results of treatment and the number of wound localizations.

**Methods:**

The treatment of 769 patients was analyzed. Pain intensity was diagnosed using a visual analog scale (VAS). To detect neuropathic pain, the Douleur Neuropathique 4 questions (DN4). The presence of an acute stress reaction (ASR) was diagnosed using The hospital anxiety and depression scale (HADS) and medical history, the diagnosis was established by a psychiatrist. Satisfaction with treatment results was studied using the Chaban quality of life scale (CQLS). Group comparisons were made using the Mann–Whitney test and the chi-square test, taking into account continuity correction.

**Results:**

Chronic pain was observed in 538 (70% 95% CI 66.7%-73.1%) patients with gunshot wounds: of them, 439 patients had wounds in 1, 2 anatomical parts of the body, here the frequency of pain chronicity is 69.7% (95% CI 66.0%-78.5%), and 99 patients had wounds in 3 or more anatomical parts of the body – 71.2% (95%CI 63.4%-78.5%). DN4 data suggest the presence of a neuropathic pain component in these patients. Also, all patients were diagnosed with ASR upon admission: the number of HADS points ranged from 9 to 25 points. CQLS data indicate that satisfaction with treatment outcomes was high (76 points) before hospital discharge, but subsequently decreased to a low level (64 points).

**Conclusions:**

Patients with gunshot wounds have a high risk of chronic pain, averaging 45% higher than the general population in civilian trauma patients. A greater frequency of the neuropathic component of pain and acute stress reactions is the reason for such chronicity. A decrease in the level of satisfaction with the results of treatment, in the remote period of observation, compared to the level at the time of discharge from the hospital, is probably a consequence of the formation of chronic pain.

**Trial registration:**

ClinicalTrials.gov: Retrospectively registered on August 1, 2022, NCT05489029.

## Background

Associating pain sensations with events in which the patient was injured is one of the strongest factors that has a psychological impact on a person. War and the conditions in which the wound was received—the conditions in which pain occurs are a key component of its chronicity [1–3]. A gunshot wound received during war is a big problem in the long-term perspective of treatment results [1, 4–6]. It is pain and memories of combat conditions, memories and pain that cause the development of states with self-destructive behavior [1, 7]. According to statistics, gunshot wounds account for 54–70% [4, 8, 9]. Gunshot wounds to the chest during ATO/OJF make up 7.4–11.7%, shrapnel wounds prevail here – 72.2%, explosive wounds – 17.5%, bullet wounds – 10.3%, and lethality – 12.2–25% [10–13]. According to the data of the Command of the Medical Forces of the Armed Forces of Ukraine, 64% of gunshot injuries occur in the structure [7, 14]. In civilian life, from 11 to 40% of patients suffer from chronic pain after receiving injuries. There are almost no data on chronic pain in patients with gunshot wounds, isolated clinical cases have been published.

Studying the specifics of pain in patients with gunshot wounds requires in-depth research, because the subjective feelings and emotional experiences experienced by patients during the wounding, in the conditions of combat, have their own, unique, features. Since pain becomes chronic in 70% of patients with gunshot wounds, the data from our study will play an important role in the treatment of such patients.

## Methods

The research was carried out on the basis of the National military medical clinical center "Main military clinical hospital". All patients participated in Operation Joint Forces (OJF) and received gunshot wounds during combat operations.

A retrospective analysis of disease histories for the period from 2014 to 2021 was carried out. Data collection was carried out at all stages of treatment: medical and nursing teams (within 2 days), military mobile hospitals (within 5 days), military medical clinical centers (within 7 days), during rehabilitation, within 12 months of the injury. In all patients, the anesthetic risk assessment was carried out according to the scale of the American Society of Anesthesiologists (ASA) – during admission to all stages of treatment. The basic tool for pain intensity research was the visual analog scale (VAS). The study of the neuropathic component of pain was carried out using a diagnostic questionnaire for detecting neuropathic pain Douleur Neuropathique 4 questions (DN4) [15]. Satisfaction with treatment results was studied using the Chaban quality of life scale (CQLS) [16–21].

### Data collection and extraction

The research was carried out within the framework of the accordance with the protocol on bioethical examination No. 158 of may 23, 2022, issued by the Commission on Biotic Expertise and Research Ethics of the Bogomolets National medical university, Ministry of Health of Ukraine. All study data were reflected in the patient's medical history. They are stored in the archives of the National military medical clinical center "Main military clinical hospital", Kyiv, 18 Hospitalna Street, Ukraine. The analysis of the research results was carried out in the EZR v.1.35 package (R statistical software version 3.4.3, R Foundation for Statistical Computing, Vienna, Austria).

### Statistical analysis

The analysis of the research results was carried out in the EZR v.1.35 package (R statistical software version 3.4.3, R Foundation for Statistical Computing, Vienna, Austria). The Shapito-Wilk test was used to check the distribution of quantitative indicators for normality. The law of distribution differed from the Gaussian, the median value (Me) and interquartile range (QI-QIII) were given to represent quantitative indicators, the comparison of indicators in two groups was carried out according to the Mann–Whitney test. For multiple testing correction the false discovery rate (FDR) procedure was used. To analyze the dynamics of the indicators, the Friedman test was used for related samples, the posterior comparison was carried out using the Bonferroni correction. For qualitative indicators, the absolute frequency of symptom manifestation and relative frequency (%) are presented, and for the comparison of two groups, the chi-square test was used, taking into account the correction for continuity. When conducting the analysis in all cases, the critical level of significance was taken equal to 0.05.

## Results

The study is based on our own clinical experience of treating 769 patients with gunshot wounds during hostilities. All patients were divided into two groups: Group 1 – patients with gunshot wounds with 1.2 localizations of injured anatomical parts of the body; Group 2 – patients with gunshot wounds with the number of localizations of injured anatomical parts of the body > 2. Thus, 630 patients were assigned to Group 1, and 139 patients to Group 2.

The results of the treatment were evaluated according to VAS – if after 3 months the patient feels pain, then such pain is considered chronic.

The distribution law is different from normal, the median Me and the interquartile range (QI-QIII) are presented. General characteristics of patients (see Table 1), and the frequency of cases of patients with gunshot wounds (see Table 2).

**Table 1:** General characteristics of patients (median Me and interquartile range are presented) (Q_I_-Q_III_)

Indicator	Group 1 (*n* = 630)	Group 2 (*n* = 139)	Level of significance of the difference, p
Age (years)	31 (25–39)	33 (25–39)	0.695
Height (cm)	178 (176–182)	178 (175.3–182)	0.799
Weight (kg)	80 (74–85)	78 (75–85)	0.855
Number of operations	5 (4–7)	5 (5–7)	0.423
Anesthesia duration (min)	125 (110–150)	125 (110–153.8)	0.731
Operation duration (min)	115 (95–140)	115 (100–140)	0.637

Comparisons were made using the Mann–Whitney test

During the analysis, no statistically significant difference in the age of patients in the groups was found (*p* = 0.695 according to the Mann–Whitney test). So the groups are comparable in terms of age, as well as height – *p* = 0.799, weight of patients – *p* = 0.855, number of surgical interventions performed – *p* = 0.423, average duration of anesthesia – *p* = 0.731, and average duration of operations – *p* = 0.637

**Table 2:** Frequency of cases (abs. (%)) of patients with gunshot wounds

Indicator		Group 1 (*n* = 630)	Group 2 (*n* = 139)	Level of significance of the difference, p
Gender	male	630 (100)	139 (100.0)	0.772
Anesthesia types	General anesthesia	205 (32.5)	43 (30.9)	0.810
	Regional anesthesia	212 (33.7)	45 (32.4)	
	Regional anesthesia and sedation	213 (33.8)	51 (36.7)	
ASA	2	29 (4.6)	3 (2.2)	0.411
	3	505 (80.2)	113 (81.3)	
	4	96 (15.2)	23 (16.5)	

Comparisons were made using the chi-square test, adjusted for continuity

During the analysis, no statistically significant difference of patients was found in the groups according to the assessment of the condition of the patients before the surgical intervention according to the classification of the American Society of Anesthesiologists (ASA) – *p* = 0.411. So the groups are comparable in terms of anesthetic risk, as well as gender – *p* = 0.772 and types of anesthesia – *p* = 0.810.

Intensity of pain according to VAS at admission to the stages of treatment and after analgesia (see Table 3). The distribution law differs from the normal one, the Me median and the interquartile range are presented (Q_I_-Q_III_).

**Table 3:** Data on pain intensity during VAS and after analgesia at admission to different stages of treatment in patients with gunshot wounds

Indicator (points)	Group 1 (*n* = 630)	Group 2 (*n* = 139)	Level of significance of the difference, *p*
VAS before anesthesia in medical and nursing teams	7 (7–8)	8 (7–9)	< 0.001*
VAS before anesthesia in military mobile hospitals	7 (6–7)	7 (6–7)	0.99
VAS before analgesia in military medical clinical centers	6 (4–7)	6 (5.25–7)	0.862
VAS during rehabilitation	2 (2–2)	2 (2–2)	0.002*
VAS after anesthesia in medical and nursing teams	4 (4–4)	4 (4–4)	0.083
VAS after anesthesia in military mobile hospitals	4 (4–4)	4 (4–4)	0.491
VAS after anesthesia in military medical clinical centers	2 (2–3)	3 (2–3.75)	< 0.001*

Comparisons were made using the Mann–Whitney test. * – difference is significant by the FDR method (Benjamini, Y. and Hochberg, Y., 1995)

Based on the obtained data, the following conclusion can be drawn: in the medical and nursing teams before anesthesia (on admission), the intensity of pain according to VAS in Groups 1 and 2 met the criteria of severe pain (ranged from 7 to 9 points), and a difference was observed depending on localization – in patients from Group 2, the intensity of pain according to VAS is higher than in patients from Group 1 (*p* < 0.001). After analgesia, such a difference is not observed (*p* = 0.083), but the intensity of pain in the two groups corresponds to the criterion of moderate pain (4 points). When entering the stage of treatment in military mobile hospitals, the intensity of pain according to VAS in two groups corresponds to the criteria of moderate (upper limit) and severe pain (the number of points ranges from 6 to 7). Later, after analgesia in military mobile hospitals, the intensity of pain decreased to moderate (4 points). At the stage of treatment in military medical clinical centers before analgesia (on admission), the intensity of pain according to the VAS in the 2 groups practically did not differ and meets the criterion of moderate and severe pain, here the number of points ranged from 4 to 7 – this indicates that in the absence of contraindications over pain and low effectiveness of pain treatment tactics. After analgesia at this stage, the pain intensity decreased to 4 points.

Patients with gunshot wounds, depending on the localization of the wound, at different stages of treatment, need to pay more attention to the tactics of pain treatment, because the lack of quality pain control and insufficient analgesia can have a significant impact on the long-term results of pain treatment, namely on its chronicity.

**Fig. 1:**
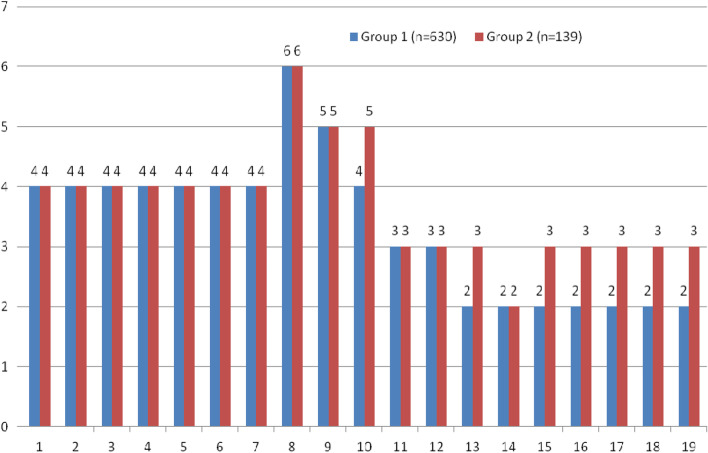
Dynamics of pain intensity according to VAS at different stages of treatment in patients with gunshot wounds: 1, 2 – stage of treatment: medical and nursing teams; 3, 4, 5, 6, 7 – stage of treatment: military mobile hospitals; 8, 9, 10, 11, 12, 13, 14, 15 – stage of treatment: military medical clinical centers; 16, 17, 18, 19 – stage of treatment: rehabilitation. Notes: Comparisons were made using the Mann–Whitney test. * – difference is significant by the FDR method (Benjamini, Y. and Hochberg, Y., 1995)

Dynamics of pain intensity according to VAS at different stages of treatment (see Fig. 1). The distribution law is different from the normal one, the median Me and the interquartile range (QI-QIII) are presented.

**Fig. 2:**
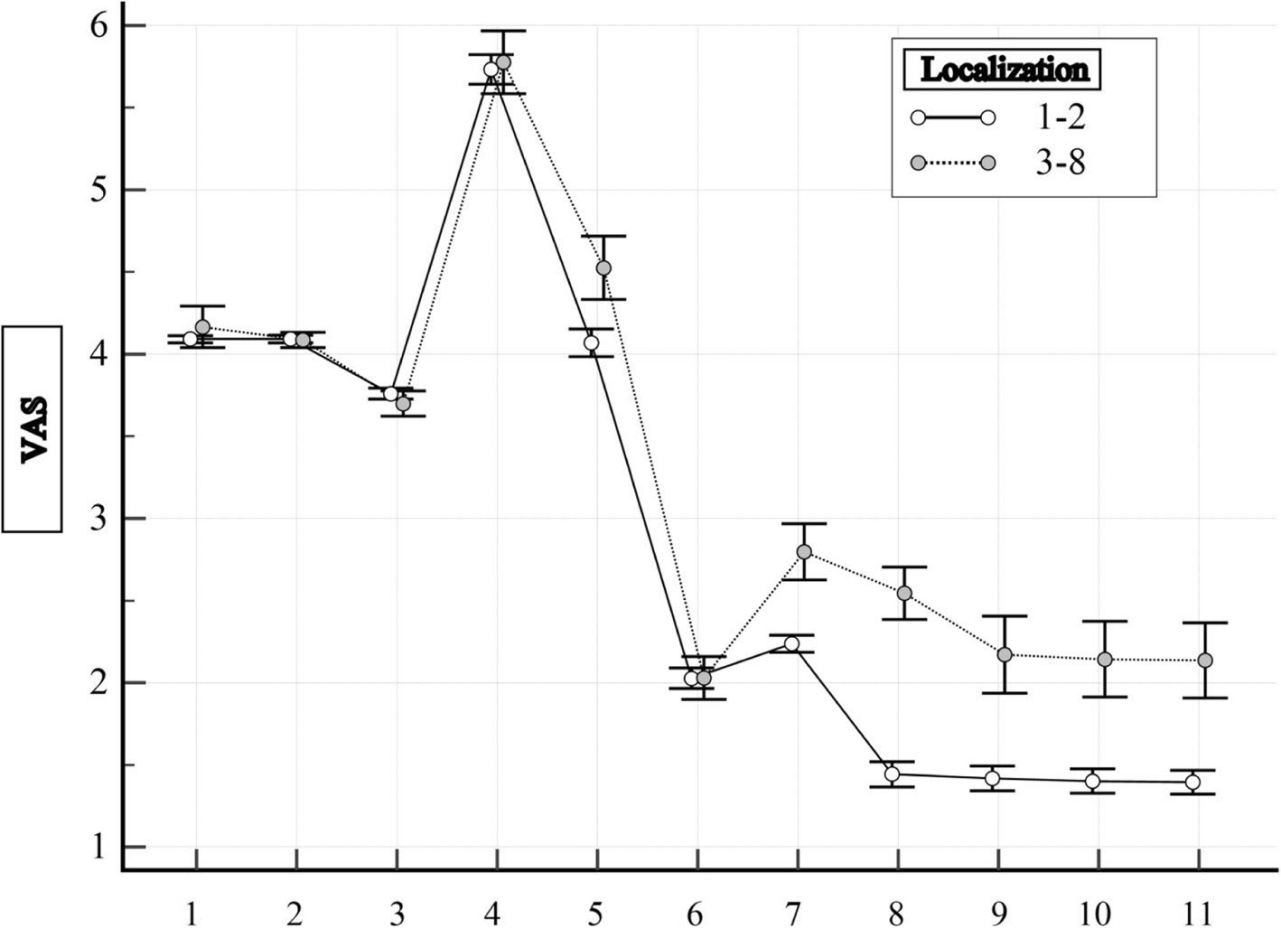
Dynamics of the VAS indicator for patients with gunshot wounds of two groups at different stages of treatment: 1 – 1 day of observation, 2 – 3 days of observation, 3 – 5 days of observation, 4 – 8 days of observation, 5 – 10 days of observation, 6 – 14 days of observation, 7 – during discharge from the military medical clinical center, 8 – 1 month after the injury, 9 – 3 months after the injury, 10 – 6 months after the injury, 11 – 12 months after the injury. The average value of the indicator and its 95% CI are indicated

From the results of the analysis, it is clear that in Groups 1 and 2 from the 1st to the 8th day of observation, the intensity of pain according to the VAS practically did not differ and ranged from 3 to 7 points, which corresponds to the criteria of moderate and severe pain: from 1 From the 7th to the 7th day of observation, the pain intensity was moderate, but on the 8th day, the pain had the highest intensity and was characterized as severe pain (see Fig. 2). During follow-up, pain intensity according to VAS decreased, but was lower in Group 1 than in Group 2 until the end of follow-up. It is worth noting that on the 14th day of observation in the 2 Groups, the intensity of pain according to the VAS practically did not differ and corresponded to 2 points (mild pain), this indicates stable control over pain and the effectiveness of pain treatment, but further observations indicate to the difference, which is most likely related to localization, that is, to the number of injured anatomical areas of the patient.

That is, in patients who received gunshot wounds with the localization of 3 or more anatomical sites, the intensity of pain according to the VAS starting from the 9th day of observation is higher than in patients who received gunshot wounds with the localization of 1 or 2 anatomical sites (see Fig. 2). 1, 3, 6, 12 months after the injury, the pain intensity index according to the VASH also differs. That is, the long-term results of pain treatment at the stages of treatment indicate that those patients who received gunshot wounds with localization of 1, 2 anatomical sites had a better outcome than patients with localization of 3 or more anatomical sites.

Analyzing the dynamics of pain intensity according to VAS in 2 groups, it can be concluded that the number of localizations of injured anatomical areas is important in predicting the tactics of pain treatment in patients with gunshot wounds.

Dynamics of intervals between analgesia at different stages of treatment (see Table 5). The distribution law differs from the normal one, the Me median and the interquartile range are presented (Q_I_-Q_III_).

**Fig. 3:**
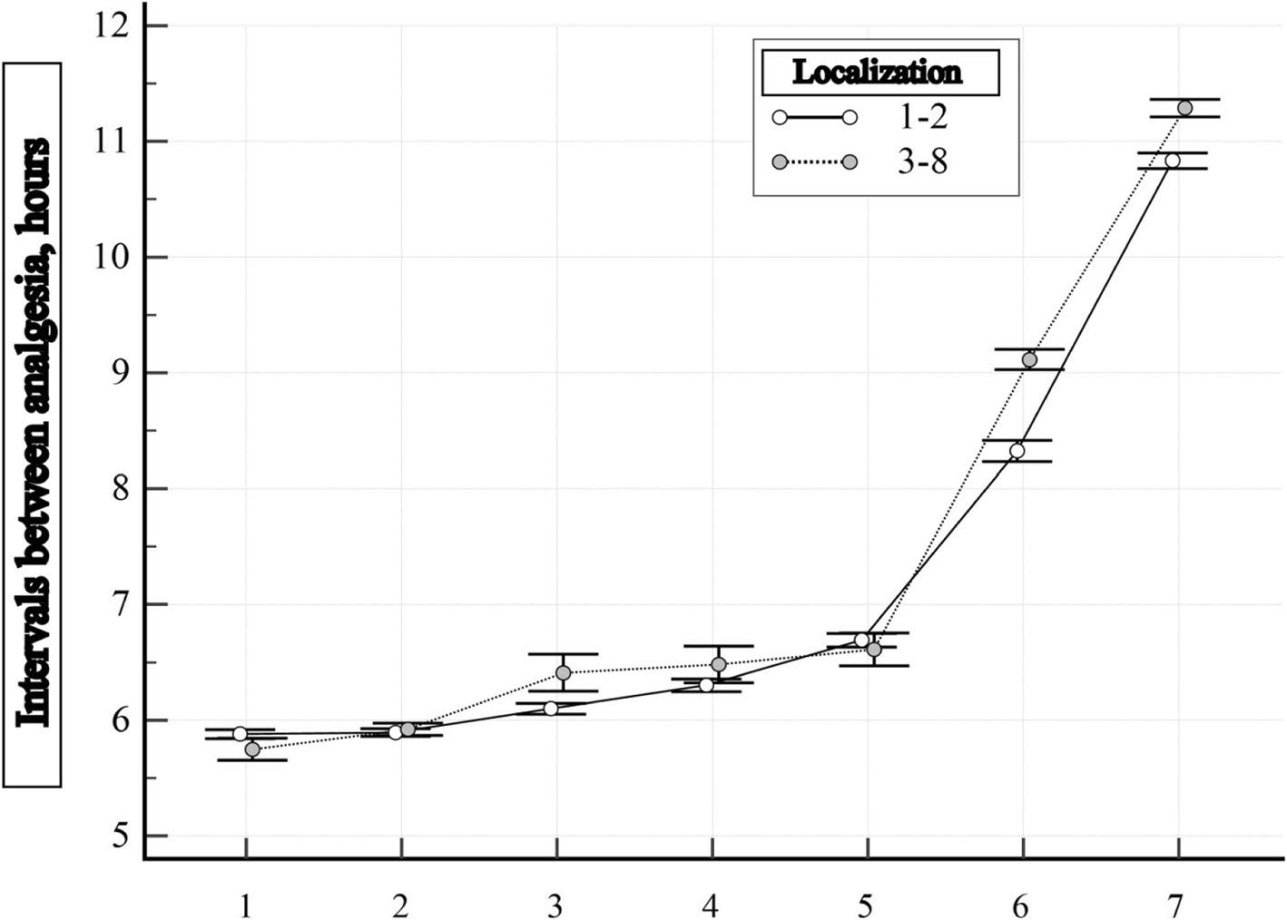
Dynamics of intervals between analgesia for patients with gunshot wounds of two groups at different stages of treatment: 1 – 1 day of observation, 2 – 3 days of observation, 3 – 5 days of observation, 4 – 7 days of observation, 5 – 9 days of observation, 6 – 11 days observation, 7–13 days of observation. The average value of the indicator and its 95% CI are indicated

From the results of the analysis, it is clear that in the 2 Groups during the 1st day of observation, upon admission at the stage of treatment in medical and nursing teams, the intervals between analgesia statistically differed (*p* = 0.001). In patients of Group 2, this interval was shorter, that is, the patients were more often administered drugs for pain relief – the average interval between pain relief was 6 h (see Fig. 3). However, on the 2nd day, this indicator did not differ in the 2 Groups (*p* = 0.818) and is an average of 6 h between analgesia (see Table 4). During the 3rd, 4th and 7th days of observation at the stage of treatment in military mobile hospitals, the intervals between analgesia practically did not differ and, on average, amounted to 6 h. On the 7th day, the interval between analgesia varied between 6 and 7 h on average, regardless of the number of wound localizations. On the 5th and 6th days of observation (stage of treatment in military mobile hospitals), an increase in the interval between analgesia is observed, there is a difference between the observation groups (*p* < 0.001). Such fluctuations in the intervals between analgesia are directly related to the number of localizations of injured anatomical parts of the body, because on the 10th, 11th, 12th, 13th and 14th days of observation, a significant increase in the intervals between analgesia can be seen on average from 8 up to 12 h, while in Group 1 the average interval between analgesia is longer than in Group 2. This indicates that the frequency of administration of drugs for analgesia depends on the number of localizations of gunshot wounds, and therefore this indicator is important for evaluating the results of pain treatment in patients with gunshot wounds.

**Table 4:** Intervals between analgesia in patients with gunshot wounds at different stages of treatment

Stage of treatment	Indicator (hours)	Group 1 (*n* = 630)	Group 2 (*n* = 139)	Level of significance of the difference, *p*
Medical and nursing teams	Interval between analgesia is 1 day of observation	6 (6–6)	6 (6–6)	0.001*
	Interval between analgesia is 2 days of observation	6 (6–6)	6 (6–6)	0.818
Military mobile hospitals	Interval between analgesia is 3 days of observation	6 (6–6)	6 (6–6)	0.483
	Interval between analgesia is 4 days of observation	6 (6–6)	6 (6–6)	0.507
	Interval between analgesia is 5 days of observation	6 (6–6)	6 (6–7)	0.004*
	Interval between analgesia is 6 days of observation	6 (6–7)	6 (6–7)	**0.034**
	Interval between analgesia is 7 days of observation	6 (6–7)	6 (6–7)	0.223
Military medical clinical centers	Interval between analgesia is 8 days of observation	6 (6–7)	6 (6–7)	0.581
	Interval between analgesia is 9 days of observation	7 (6–7)	6 (6–7)	0.079
	Interval between analgesia is 10 days of observation	8 (7–8)	7 (6–8)	< 0.001*
	Interval between analgesia is 11 days of observation	8 (8–9)	9 (9–9)	< 0.001*
	Interval between analgesia is 12 days of observation	10 (9–12)	9 (9–12)	< 0.001*
	Interval between analgesia is 13 days of observation	11 (10–12)	11 (11–12)	< 0.001*
	Interval between analgesia is 14 days of observation	12 (11–12)	11 (11–12)	< 0.001*

Comparisons were made using the Mann–Whitney test. * – difference is significant by the FDR method (Benjamini, Y. and Hochberg, Y., 1995)

Diagnosis of the neuropathic component of pain in patients with gunshot wounds (see Table 5). If the patient has 4 points or more, this indicates that a neuropathic pain component is present. The distribution law differs from the normal one, the Me median and the interquartile range are presented (Q_I_-Q_III_).

**Table 5:** The results of the diagnosis of the neuropathic component of pain in patients with gunshot wounds depending on the number of injury localizations of anatomical parts of the body at different stages of treatment during the observation period

Indicator (points)	Group 1 (*n* = 630)	Group 2 (*n* = 139)	Level of significance of the difference, *p*
DN4 during treatment in military mobile hospitals	5 (4–5)	5 (4–5)	0.937
DN4 during treatment in military medical clinical centers	5 (5–5)	5 (5–5)	0.911
DN4 during discharge from military medical clinical centers	5 (2–5)	5 (2–5)	0.933
DN4 1 month after injury	5 (2–5)	5 (2–5)	0.918
DN4 3 months after injury	5 (2–5)	5 (2–5)	0.713
DN4 6 months after injury	5 (2–5)	5 (2–5)	0.824
DN4 12 months after injury	5 (2–5)	5 (2–5)	0.911

Comparisons were made using the Mann–Whitney test

**Fig. 4:**
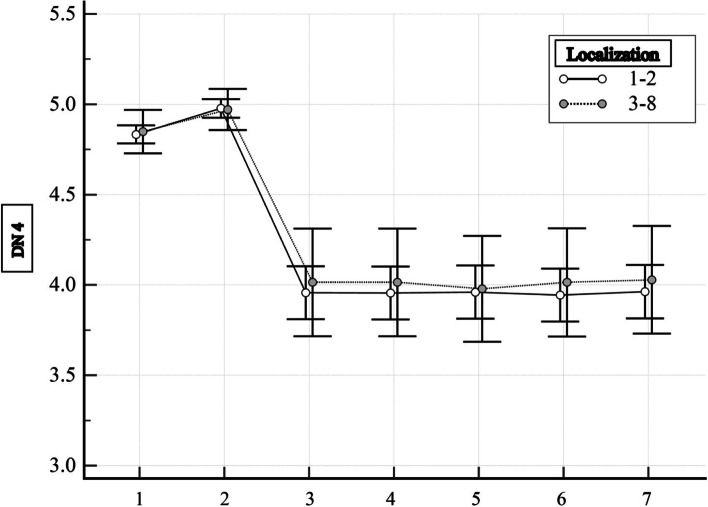
Dynamics of the DN4 indicator for patients of two groups: 1 – during treatment in military mobile hospitals, 2 – during treatment in military medical clinical centers, 3 – during discharge from military medical clinical centers, 4 – 1 month after injury, 5 – 3 months after injury, 6 – 6 months after injury, 7 – 12 months after injury. The average value of the indicator and its 95% CI are indicated

The Mann–Whitney test was used to compare two groups. From the results of the analysis, it is clear that there is no statistically significant difference at all stages of treatment, in particular when comparing at the stage of military mobile hospitals – *p* = 0.937, military medical clinical centers – *p* = 0.911. During treatment, at these stages, the indicators of the DN4 diagnostic questionnaire indicate that all patients have a neuropathic component of pain (data range from 4 to 5 points). However, at the time of discharge at the stage of treatment in military medical clinical centers, the absence of a neuropathic component of pain is observed in some patients (diagnostic questionnaire DN4 data > 4 points). There is also no statistically significant difference between the observation groups—*p* = 0.933. Further, 1 month – *p* = 0.918, 3 months – *p* = 0.713, 6 months – *p* = 0.824 and 12 months – *p* = 0.911 follow-up, and the DN4 diagnostic questionnaire data indicated that this indicator did not differ. Figure 4 shows the dynamics of the DN4 indicator for patients of two groups. It can be seen here that this indicator had the highest value at the time of admission to the military medical clinical centers, then it decreased at the time of discharge, and thereafter it practically did not change during the entire observation period. However, data throughout the observation period indicate the presence of a neuropathic component of pain. This suggests that the likelihood of a negative pain treatment outcome in patients with gunshot wounds is related to the presence of a neuropathic component.

Assessment of the level of satisfaction with treatment results (see Table 6). The distribution law differs from the normal one, the Me median and the interquartile range are presented (Q_I_-Q_III_).

**Table 6:** Satisfaction with treatment outcomes in patients with gunshot wounds during follow-up

Indicator (points)	Group 1 (*n* = 630)	Group 2 (*n* = 139)	Level of significance of the difference, *p*
Level of satisfaction with the results of treatment before discharge from military medical clinical centers	73 (68–76)	73 (68–76)	0.913
Level of satisfaction with treatment results 1 month after injury	64 (64–68)	64 (64–68)	0.805
Level of satisfaction with treatment results 3 months after injury	64 (64–68)	64 (64–68)	0.845
Level of satisfaction with treatment results 6 months after injury	64 (64–68)	64 (64–68)	0.851
Level of satisfaction with treatment results 12 months after injury	64 (64–68)	64 (64–68)	0.752

Comparisons were made using the Mann–Whitney test

The Mann–Whitney test was used to compare two groups. Table 6 shows that the level of satisfaction of patients with gunshot wounds before discharge from military medical clinical centers in Group 1 ranged from 68–76 points (average value – 73 points), in Group 2 – 68–76 points (average value – 73 points) – *p* = 0.913; after 1 month, this indicator in Group 1 and 2 is from 64 to 68 points (average value – 64 points) – *p* = 0.845; after 3 months, this indicator in Group 1 and 2 is from 64 to 68 points (average value – 64 points) – *p* = 0.851; after 12 months, this indicator in Group 1 and 2 is from 64 to 68 points (average value – 64 points) – *p* = 0.752, that is, there is no statistically significant difference between the groups.

Analyzing the dynamics of the assessment of the level of satisfaction with the results of treatment in patients with gunshot wounds, it is clear that the maximum level of satisfaction with the results of treatment in patients of the two groups during the entire observation period was before discharge from inpatient treatment and ranged from 68 – the average level of satisfaction to 76 points – this corresponds to high level of satisfaction. Later, after 1, 3, 6 and 12 months of observation, this indicator decreased and ranged from 64 to 68 pains, which corresponds to the average level of satisfaction with the treatment results. Taking into account that the level of satisfaction with the results of treatment depends partly on the patient's psychological status, unpleasant sensations in the anatomical area of the injury and emotional experiences that are associated with the events and circumstances in which the patient was injured, such results indicate the probability of the influence of chronic pain on level of satisfaction with treatment results.

## Discussion

In civilian life, from 11 to 40% of patients suffer from chronic pain after receiving injuries. Studies on chronic pain in patients with gunshot wounds are very few and are presented in isolated cases. The experience gained by military doctors in Ukraine is unique and aimed at improving the results of pain treatment in such patients.

As a result of our study, it was found that chronic pain was observed in 538 of 769 patients (70% 95% CI 66.7%-73.1%). Those patients who were wounded in 3 or more anatomical parts of the body had a higher percentage of chronic pain—71.2% (95% CI 63.4%-78.5%) than in patients with gunshot wounds in 1 or 2 anatomical parts of the body, here the frequency of chronic pain was 69.7% (95% CI 66.0%-78.5%). That is, the probability of chronic pain increases in patients who received gunshot wounds in a larger number of anatomical parts of the body.

DN4 data on admission to military medical clinical centers indicate the presence of a neuropathic component of pain in such patients, suggesting that the likelihood of receiving a negative result of pain treatment in patients with gunshot wounds is associated with the presence of a neuropathic component of pain.

All patients were diagnosed with HSR upon admission: the number of HADS points ranged from 9 to 25 points. The level of satisfaction with the results of treatment according to the CQLS corresponded to the average level.This indicator depends on the psychological status of the patient, sensations in the injured anatomical part of the body and emotional experiences that are associated with the events and circumstances in which the patient was injured—here the results indicate the probability of the influence of chronic pain on the level of satisfaction with the results of treatment. CQLS data indicate that satisfaction with treatment outcomes was high (76 points) before hospital discharge, but subsequently declined to a low level (64 points).

The problem of chronic pain in patients with gunshot wounds requires even more in-depth study, therefore, further research aimed at studying predictors of the negative outcome of pain treatment in this category of patients will have significant scientific significance, because the subjective feelings and emotional experiences experienced by patients during wounds in combat have their own unique characteristics.

## Conclusions

An analysis of pain outcomes in 769 patients with gunshot wounds in a combat setting showed a high risk of pain chronicity, an average of 45% greater than the general population in civilian injured patients. A greater frequency of the neuropathic component of pain and acute stress reactions is the reason for such chronicity. A decrease in the level of satisfaction with the results of treatment, in the remote period of observation, compared to the level at the time of discharge from the hospital, is probably a consequence of the formation of chronic pain.

## Data Availability

The datasets used and analysed during the current study are available from the corresponding author on reasonable request.
